# The interplay of inflammation, exosomes and Ca^2+^ dynamics in diabetic cardiomyopathy

**DOI:** 10.1186/s12933-023-01755-1

**Published:** 2023-02-20

**Authors:** Santosh K Sanganalmath, Shubham Dubey, Sudhakar Veeranki, Keerthy Narisetty, Prasanna Krishnamurthy

**Affiliations:** 1 Department of Internal Medicine, Division of Cardiovascular Medicine, University of Nevada Las Vegas School of Medicine, Las Vegas, NV 89102 USA; 2 Department of Biomedical Engineering, Schools of Medicine and Engineering, University of Alabama at Birmingham, University Blvd., Birmingham, AL 35294 USA; 3 Department of Molecular and Cellular Biochemistry, University of Kentucky, Lexington, KY 40506 USA; 4 Advanced Heart Failure Management Center, Edgewood, KY 41017 USA

**Keywords:** Calcium signaling, Diabetic cardiomyopathy, Exosome, Heart failure, Inflammation, Mitochondrial membrane

## Abstract

Diabetes mellitus is one of the prime risk factors for cardiovascular complications and is linked with high morbidity and mortality. Diabetic cardiomyopathy (DCM) often manifests as reduced cardiac contractility, myocardial fibrosis, diastolic dysfunction, and chronic heart failure. Inflammation, changes in calcium (Ca^2+^) handling and cardiomyocyte loss are often implicated in the development and progression of DCM. Although the existence of DCM was established nearly four decades ago, the exact mechanisms underlying this disease pathophysiology is constantly evolving. Furthermore, the complex pathophysiology of DCM is linked with exosomes, which has recently shown to facilitate intercellular (cell-to-cell) communication through biomolecules such as micro RNA (miRNA), proteins, enzymes, cell surface receptors, growth factors, cytokines, and lipids. Inflammatory response and Ca^2+^ signaling are interrelated and DCM  has been known to adversely affect many of these signaling molecules either qualitatively and/or quantitatively. In this literature review, we have demonstrated that Ca^2+^ regulators are tightly controlled at different molecular and cellular levels during various biological processes in the heart. Inflammatory mediators, miRNA and exosomes are shown to interact with these regulators, however how these mediators are linked to Ca^2+^ handling during DCM pathogenesis remains elusive. Thus, further investigations are needed to understand the mechanisms to restore cardiac Ca^2+^ homeostasis and function, and to serve as potential therapeutic targets in the treatment of DCM.

## Introduction

**Fig. 1:**
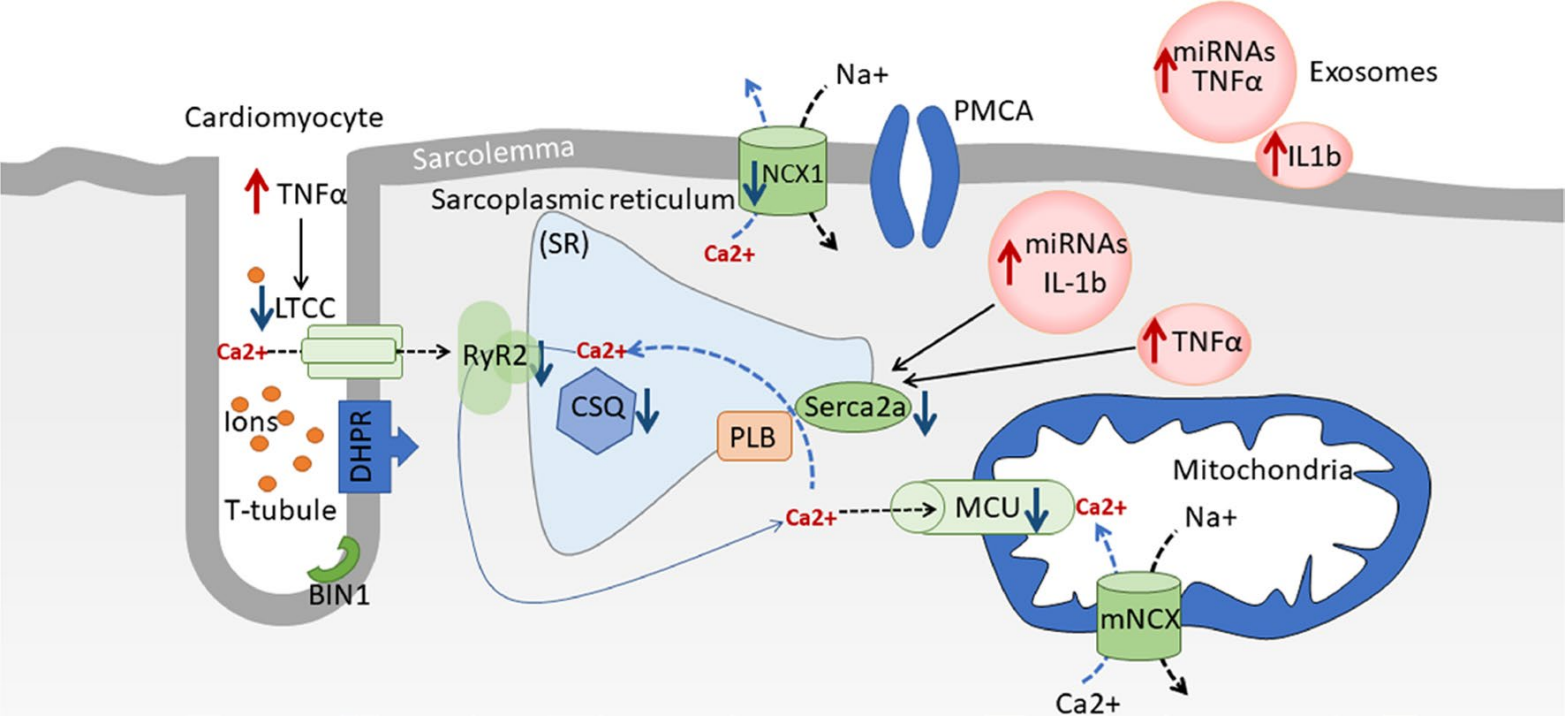
Potential effects of inflammation,  exosomes, and microRNA (miRNA) on Ca^2+^ transport, storage and mitochondrial Ca^2+^ handling. Excitation–contraction (EC) coupling is initiated by an action potential which depolarizes the sarcolemma by rapid sodium (Na^+^) influx. Depolarization activates voltage-gated L-type Ca^2+^ channels (LTCC), and Ca^2+^ influx triggering calcium-induced calcium release (CICR) from the sarcoplasmic reticulum (SR) via the ryanodine receptor (RyR2). Rapid release of Ca^2+^ from the SR increases free intracellular Ca^2+^, enabling muscle contraction. Cardiomyocyte relaxation is regulated by signaling pathways that restore intracellular and SR Ca^2+^ to resting concentrations. Ca^2+^-activated kinases phosphorylate phospholamban (PLB), relieving its repression on Sarco(endo)plasmic reticulum Ca^2+^-ATPase (SERCA2a). Consequently, SERCA2a rapidly imports Ca^2+^ into the SR, decreasing the intracellular Ca^2+^ concentration. Na^+^/Ca^2+^ exchangers (NCX) are allosterically activated by Ca^2+^ and aid in restoring resting Ca^2+^ concentrations; decreased cytosolic Ca^2+^ leads to relaxation of the sarcomere. Genes downregulated in DCM are denoted by a blue downward arrow and genes upregulated during DCM are denoted by a red upward arrow. Mitochondria is an energy mobilization and  Ca^2+^-buffering organelle. The Ca^2+^ homeostasis is controlled by its uptake through the mitochondrial Ca^2+^ uniporter (MCU) complex and voltage-dependent channel proteins, Ca^2+^ efflux is controlled by NCX. Exosomes and miRNAs control the gene expression of certain inflammatory cytokines, Ca^2+^ handling and signaling proteins. *DHPR* Dihydropyridine receptor; *BIN1* bridging integrator 1; *PMCA* Sarcolemmal/plasma membrane Ca^2+^-ATPase; *CSQ* calsequestrin, *mNCX* Mitochondrial N^+^/Ca^2+^ exchanger; *TNF-α* Tumor necrosis factor-α; *IL1b* Interleukin 1β

Diabetic cardiomyopathy (DCM) is one of the end-stage consequences of mortality and morbidity in patients with diabetes mellitus. Diabetes stimulates chronic inflammation, alters Ca^2+^ homeostasis, activates cardiac fibroblast transformation into myofibroblast leading to left ventricular dysfunction and worsening clinical outcomes [1, 2]. Cardiac function is partly dependent on the rhythmic contractions of cardiac muscle, which incessantly goes through contraction and relaxation cycles. As cardiac contractility is regulated by the intracellular calcium concentrations [Ca^2+^]_i_, which also changes during contraction/relaxation (systolic and diastolic) cycles, the regulators of [Ca^2+^]_i_ are the major determinants of cardiac function. In the ventricular myocyte, Ca^2+^ moves around the sarcoplasmic reticulum, mitochondrial membrane and sarcolemma through different ion channels and ion transporters (Fig. 1). During myocardial excitation–contraction (EC) coupling, extracellular Ca^2+^ moves into the cardiomyocyte via L-type voltage-dependent Ca^2+^ channels (LTCC) and reverse sodium-calcium (Na^+^/Ca^2+^) exchanger [3, 4]. This influx of Ca^2+^ serves as a trigger and induces release of Ca^2+^ from the sarcoplasmic reticulum (intracellular Ca^2+^ store), through the ryanodine receptors (RyR2), a process known as calcium-induced calcium release (CICR) [5, 6]. This sudden availability of cytosolic free Ca^2+^ in large amounts results in simultaneous Ca^2+^ binding to multiple cardiac troponin C molecules, which is part of the troponin complex attached to the thin filament that regulates myosin heavy chain (MHC) filament binding to the actin in the thin filament. When Ca^2+^ binds to troponin C, it results in activation of myofilaments eventually leading to contraction**.** Cytosolic Ca^2+^ concentration must decline before the occurrence of relaxation and diastolic filling. Therefore, as soon as cytosolic Ca^2+^ dissociates from troponin C, the Ca^2+^ is cleared from the cytosol leading to the termination of contraction. Four different transporters remove Ca^2+^ from the cytosol: (i) the sarcoplasmic reticulum Ca^2+^-ATPase (SERCA2a), (ii) sarcolemmal Na^+^/Ca^2+^ exchanger, (iii) sarcolemmal/plasma membrane Ca^2+^-ATPase, and (iv) the mitochondrial Ca^2+^ uniporter (MCU).

Pathophysiological alterations during the early stages of DCM involve asymptomatic left ventricular dysfunction with a near normal ejection fraction, which eventually progresses to impaired cardiac contractility and detrimental arrhythmias. Table 1 summarizes  cardiac structural and functional changes observed in clinical studies and in different animal models in two forms of diabetes mellitus (insulin-dependent diabetes or Type I and insulin-resistance diabetes or Type II). Given the multitude of Ca^2+^ handling proteins governing the Ca^2+^ transients in the cytosol of the cardiomyocyte, DCM has been known to adversely affect many of these Ca^2+^ handling proteins either qualitatively and/or quantitatively [7]. These changes are either adaptive or maladaptive in nature depending on the stage of the DCM. The etiology and pathophysiology of diabetes is very complex in nature, which is also reflected in contradicting observations made by various researchers, who have studied Ca^2+^ handling and subcellular organelle remodeling in DCM. Interestingly, disturbances in Ca^2+^ homeostasis is noticeable during the early stages of DCM, further emphasizing the detrimental role of Ca^2+^ in the pathophysiology of the DCM [8]. As demonstrated by Pereira et al. in the obese model of diabetes [9], defective Ca^2+^ influx through reduced LTCC resulted in reduced stimulation of Ca^2+^ currents, which further led to reduced Ca^2+^release from the endoplasmic reticulum. In addition to the attenuated Ca^2+^ current trigger, they showed chronic suppression of sarcoplasmic reticulum Ca^2+^ load due to the inhibition of SERCA2a leading to sarcoplasmic reticulum Ca^2+^ uptake and enhanced efflux of Ca^2+^ through Na^+^/Ca^2+^ exchanger, culminating in chronically reduced sarcoplasmic reticulum Ca^2+^ load leading to Ca^2+^ moving out of the cardiomyocytes.

**Table 1:** Studies in animal models and humans with type I and/or type II diabetes showing cardiac structural and functional abnormalities

Study model	Cardiac functional changes	Diabetes type	References
Humans	↑ HR, ↑ LV wall thickness	Type II	Galderisi et al. [244]
NZ diabetic rabbit	↑ Susceptibility to ischemia	Type I	Hadour et al. [245]
STZ-induced rat	LV systolic and diastolic dysfunction	Type I	Joffe et al. [246]
Humans	↑ LV mass and wall thickness, ↓ cardiac function	Type II	Devereux et al. [134]
Isolated perfused heart of db/db mouse	↓ Cardiac contractility, ↓ Glucose oxidation	Type II	Belke et al. [247]
Humans	↑ LV thickness and mass, ↑ cardiac output	IGT	Ilercil et al. [248]
Humans	↑ LV thickness and mass	Type I	Carugo et al. [249]
IGF-1 transgenic mice	Systolic and diastolic dysfunction, ↓ LV compliance	Type I	Kajstura et al. [250]
Humans	Diastolic dysfunction, Normal systolic function	Type I	Schannwell et al. [251]
Diabetic apoB transgenic mice	Diastolic and systolic dysfunction, ↑ BNP	Type I	Nielsen et al. [252]
Non-obese diabetic mice and rat	Systolic and diastolic dysfunction, ↓ Contractility	Type I	Pacher et al. [253]
db/db and transgenic db/db-hGLUT4 mice	Systolic and diastolic dysfunction, ↓ Contractility	Type II	Semeniuk et al. [254]
Isolated perfused heart of STZ-induced diabetic mice	Systolic dysfunction, ↓ Contractility	Type I	Trost et al. [85]
Isolated perfused heart of Zucker fatty rats	Systolic and diastolic dysfunction	Type II	Young et al. [255]
Isolated perfused heart of db/db mice	↑ Susceptibility to ischemia and dysfunction	Type II	Aasum et al. [256]
db/db and ob/ob mice	Myocyte hypertrophy	Type II	Barouch et al. [257]
ob/ob mice	Diastolic dysfunction	Type II	Christoffersen et al. [258]
Humans	LVH, diastolic dysfunction	Type II	Bell [259]
db/db mice	Contractile dysfunction	Type II	Belke et al. [44]
Zucker fatty mice	LVH	Type II	Conti et al. [260]
Goto-Kakizaki diabetic rat	LVH	Type II	Desrois et al. [261]
Zucker fatty mice and Goto-Kakizaki rat	↑ Susceptibility to ischemia	Type II	Kristiansen et al. [262]
Isolated perfused heart of ob/ob mice	LVH	Type II	Mazumder et al. [263]
Biobreeding diabetic rat	↓ HR, ↓ contractility	Type I	Broderick and Hutchison [264]
ob/ob mice	LVH, ↓ ionotropic response	Type II	Boudina et al. [265]
Biobreeding diabetic rat	Diastolic dysfunction, ↓ HR, ↓ contractility	Type I	Broderick and Poirier [266]
ob/ob mice and db/db mice	↓ Contractility	Type II	Buchanan et al. [267]
db/db mice	Cardiomyocyte dysfunction	Type II and I	Kralik et al. [268]
Sucrose-fed rats	Diastolic and systolic dysfunction	Type II	Vasanji et al. [269]
STZ-induced transgenic mice	Contractile dysfunction	Type I	Suarez et al. [270]
Genetic (Akita) mice	Diastolic dysfunction	Type I	Basu et al. [271]
db/db mice	Reduced stress compliance	Type II	Daniels et al. [272]
STZ-induced rats	Increased stiffness	Type I	Bupha-Intr et al. [273]
Zucker fatty rats	Impaired contraction	Type II	Howarth et al. [274]
Genetic (Akita) mice	Diastolic and systolic dysfunction	Type I	Patel et al. [275]
Goto-Kakizaki rat	Systolic dysfunction	Type II	Salem et al. [276]
Otsuka Long-Evans Tokushima Fatty rats	Diastolic dysfunction	Type II	Takada et al. [277]
STZ-induced rats	Contractile dysfunction	Type I	Ward et al. [80]
STZ-induced mice	Diastolic dysfunction with Ca^2+^ overload	Type I	Namekata et al. [278]
Humans	Diastolic dysfunction	Type II	Lamberts et al. [279]
Humans	Impaired LV midwall shortening	Type II	Cioffi et al. [280]
Humans	Contractile dysfunction	Type II	Montaigne et al. [281]
STZ-induced transgenic mice	Diastolic and systolic dysfunction	Type I	Thomas et al. [282]
STZ-induced guinea pig	Impaired contraction after stress	Type I	Tocchetti et al. [283]
db/db mice	Contractile dysfunction	Type II	Veeranki et al. [119]
STZ-induced mice	Diastolic and systolic dysfunction	Type I	Ruiz et al. [284]

*NZ* New Zealand; *IGF-1* Insulin growth factor-1; *GLUT* Glucose transporter; *HR* Heart Rate; *LV* Left Ventricular; *BNP* B-Type natriuretic peptide; *NOD* Non-obese diabetic; *STZ* Streptozotocin; *LVH* Left ventricular hypertrophy; *ob/ob* obese mouse

Myocardial inflammation is one of the contributing factors for the development of DCM triggering many inflammatory signaling pathways. Moreover, abnormalities in Ca^2+^ homeostasis is involved in pathogenesis of cardiac inflammation that could be related to the increased Ca^2+^ signals and inflammatory responses [10]. Several proinflammatory cytokines, such as TNF-α, IL6, IL8, IL1β, and IL1 and other molecules such as IFNγ, chemokines (MCP-1, IL8 and biglycan) actively contribute to the myocardial oxidative stress, fibrosis, and cardiac dysfunction [11]. However, the effect of inflammation on Ca^2+^ signaling in DCM needs further investigation.

While the interplay of heart disease and diabetes is very complex, recent reports show that extracellular vesicles such as exosomes play a crucial role in the pathophysiology of DCM as well. Exosomes are nanosized vesicles which contain different types of cargo molecules: mRNAs, DNAs, proteins, lipids, miRNAs, released by the fusion of multivesicular body with the cell membrane [12]. Their function solely depends upon the origin of cell/tissue, and they play a critical role in angiogenesis, inflammation, and coagulation. Their beneficial role has been explored in various pathophysiological processes such as improving cardiac function, mitigating inflammatory response, and regulating immune responses [13].

In diabetes, structural composition and exosome cargo are modified as the original cells are altered by the diabetic milieu [14]. Recent studies demonstrate the role of heat shock protein 20 (Hsp20) in increasing the production of cardiomyocyte exosomes by interacting with Tumor Susceptibility 101 (TSG101), suggesting the contribution of pathogenic exosomes in the development of DCM [15]. Although there are few reports that suggest the role of exosome-mediated cellular communication in DCM, their actual role in pathophysiology of DCM remains unknown.

## Rationale

In last couple of decades, numerous studies have been conducted in the space of DCM, but very limited information is available about the effect of Ca^2+^ signaling in DCM. In this review, we discuss the remodeling of subcellular organelles in DCM and how this remodeling potentially contributes to disruption in Ca^2+^ dynamics/homeostasis. We also review the effect of inflammation and potential role of exosomes in Ca^2+^ signaling and its impact on pathogenesis of DCM. Furthermore, we also briefly discuss the role of miRNA and its regulation of Ca^2+^ signaling in DCM.

## Literature review methodology

The systematic search for all recent relevant literature was done using Pubmed (https://www.ncbi.nlm.nih.gov/PubMed), Google scholar (https://www.scholar.google.com) with the keywords such as Ca^2+^ signaling, EC coupling, diabetes-associated cardiomyopathy, myocardial inflammation, exosomes, Ca^2+^ reflux, sarcolemma, sarcoplasmic reticulum, Ca^2+^ binding proteins, effect of inflammation on Ca^2+^, and changes in mitochondria and extracellular matrix (ECM). Thorough screening of titles and abstracts was done to see the potential relevance. After identifying the deemed relevancy, full-fledged papers were reviewed in-detail to be considered for inclusion. English was the only publication language considered by the authors. Articles that were not peer-reviewed, were excluded, and were not considered. Additionally, we focused on articles published in the last 20 years and the selection was made based on their citation frequency. All the articles cited throughout the manuscript have been mentioned in the reference section along with their journal information.

## I. DCM prevalence and risk factors

Diabetes mellitus is associated with increased cardiovascular complications, including hypertension, coronary artery disease and heart failure [16, 17]. However, there is increasing evidence of association of diabetes in development of primary myocardial disease known as DCM, characterized by abnormal myocardial structure, dilated, and impaired contraction of ventricles [18, 19]. Large population-based data show that heart failure occurs in approximately 19–26% of patients with diabetes [20, 21]. Moreover, studies have demonstrated an increase in the rate of heart failure is independent of other comorbidities such as obesity, hypertension, and other types of heart diseases [22, 23]. Furthermore, the data from Cardiovascular Health Study suggests detrimental cardiac remodeling in diabetic patients, as evidenced by increase in left ventricular mass and left ventricular wall thickness with diastolic and systolic dysfunction compared to normal individuals [24–26].

DCM can occur at any age, it can occur in children, adults and elderly [27]. The patients affected by DCM could present with various symptoms, including asymptomatic cardiomegaly, sudden death, peripheral edema and orthopnea [28].

DCM prevalence is increasing parallelly with the increase in diabetes mellitus. Diabetes is a complex disease characterized by impaired cardiac function because of imbalance in antioxidants and pro-oxidants at the cellular level. Also, high sugar diet induces cardiomyocyte autophagy, oxidative stress and fibrosis [29]. Although large evidence initially reported detrimental structural changes in the heart in diabetic patients with concomitant obesity and hypertension [30], studies have shown Type II diabetes mellitus independently increases left ventricular mass and causes detrimental cardiac remodeling by itself [31].

## II. Sarcolemmal changes in DCM

### IIa. L-type  Ca^2+^ channels (LTCC)

The LTCC, CaV1.2 plays a key role in the initiation of the Ca^2+^ currents to kickstart the EC coupling [32, 33]. Therefore, the level of Ca^2+^ current generated eventually determines the intensity of the CICR and contributes to the extent of contractile force generated. It was shown that DCM due to both Type I and Type II diabetes involves contractile impairment. Consistent with this impairment, it was noted that the Ca^2+^ currents generated by the LTCC were also reduced. In the case of Type I diabetes, the surface density of this channel was reduced owing to the decreased trafficking to the cell surface [33]. In addition to lowered trafficking to the cell surface, the expression levels of CaV1.2 was also reduced in Type II diabetic mouse models [33]. Moreover, reports suggested that decline in caveolin-3 (Cav3), a known interactor of LTCC and organizer of the macromolecular complexes in the caveolae, may also contribute to the reduced presence of LTCC at the T-tubular membrane [8, 34]. Alternatively, it is possible that Cav3 depletion might have diminished the LTCC interactions with other signal transducers leading to diminished function [35]. Furthermore, CaV1.2, Ras-related G-proteins were also reported to interact with the LTCC and modulate its trafficking or function [36, 37]. However, the functional relevance of such interactions in DCM is not clear. Interestingly, it was demonstrated that LTCC negatively auto-regulates its expression through its truncated c-terminal fragment through a feedback mechanism [38]. It is unclear whether the lower LTCC expression in Type II diabetes is due to the negative after effects of a failed compensatory mechanism in response to the lack of sufficient contractile force (due to mounting demand).

### IIb. Na^+^/Ca^2+^ exchanger

Although Na^+^/Ca^2+^ exchanger can function as a bidirectional Ca^2+^ pump, under normal physiological conditions, Na^+^/Ca^2+^ exchanger serves as the main Ca^2+^ extruder at the end of the EC coupling cycle [39]. Its dysfunction or depletion leads to Ca^2+^ overloading and contributes as one of the pathophysiologic mechanisms in DCM [40]. The diabetes-mediated regulation of Na^+^/Ca^2+^ exchanger activity is complicated, and accumulated evidence suggests that diabetes may increase or decrease or do not change the Na^+^/Ca^2+^ exchanger activity. Schaffer et al. in their experiments using insulin-independent diabetic rat (a condition generated by the injection of streptozotocin) has shown a decrease in cardiomyocyte sarcolemmal Na^+^/Ca^2+^ exchanger activity without affecting the mRNA levels [41]. In contrast to this report, Hattori et al. suggested that both the Na^+^/Ca^2+^ exchanger activity and mRNA levels were reduced in diabetes, while insulin supplementation reversed these effects [42]. This indicates that the lower Na^+^/Ca^2+^ exchanger function observed in diabetic myocytes maybe in part due to the quantitative decrease in Na^+^/Ca^2+^ exchanger machinery through decreased expression [42]. A recent study reported that Na^+^/Ca^2+^ exchanger levels were in fact elevated in the genetic models of Type I diabetic hearts [43]. However, in the obese diabetic models (db/db mice), the Na^+^/Ca^2+^ exchanger activity was not significantly altered [44]. Therefore, to reconcile the differences in observed effects, it might be worth determining if diabetes alters Na^+^/Ca^2+^ exchanger activity through epigenetic mechanisms in different diabetic models. In this context, diabetes was shown to enhance miRNA that target Na^+^/Ca^2+^ exchanger [45, 46]. Furthermore, changes in acetylation of transcription factor by histone deacetylases (HDACs) also contributes to alterations in the expression of Na^+^/Ca^2+^ exchanger at the transcriptional level [47]. Such regulation might play an important role in differential expression of Na^+^/Ca^2+^ exchanger in different diabetic models. Additional complexity might also exist, as it was demonstrated that diminished SERCA2a function also leads to elevations in the Na^+^/Ca^2+^ exchanger activity through Ca^2+^/calmodulin-dependent protein kinase (CaMK)/PKB/FoxO3a/miR-1 pathway and may lead to further deterioration of cardiac function in diabetes [48].

Direct post-translational modifications of Na^+^/Ca^2+^ exchanger may also regulate its activity. Consistent with this hypothesis, Na^+^/Ca^2+^ exchanger has been shown to form macromolecular complexes at its large intracellular loop, consisting of PKA, PKC and phosphatases (PP1 and PP2A). The complex seems to regulate phosphorylation status of Na^+^/Ca^2+^ exchanger and presumably its activity [49–51]. The possibility of existence of macromolecular complex with kinases and phosphatases suggest highly dynamic regulation of Na^+^/Ca^2+^ exchanger activity in relation to the [Ca^2+^]_i_ levels. Such dynamic regulation is necessary, as Na^+^/Ca^2+^ exchanger is one of the major Ca^2+^ extruder channels, which regulate cardiomyocyte contraction ability and pathologic responses. Though the direct phosphorylation status was not assessed, it was shown that enhanced PKC activity was associated with the decline in Na^+^/Ca^2+^ exchanger activity in diabetes [52]. Nonetheless, the role of direct phosphorylation of Na^+^/Ca^2+^ exchanger and its effect on its activity during diabetes remains elusive. In summary, the decrease in Na^+^/Ca^2+^ exchanger activity observed in diabetic myocytes may be in part due to the quantitative decrease in functional Na^+^/Ca^2+^ exchanger machinery and decreased expression. However, other possible mechanisms might include reduced activation of PKCα and/or transfer of PKCβ and compositional changes in cell membrane phospholipids.

### IIc. Sarcolemmal/Plasma membrane Ca^2+^-ATPase

The plasma membrane Ca^2+^-ATPase pump plays a less critical role in extrusion of the [Ca^2+^]_i_ when compared to the Na^+^/Ca^2+^ exchanger. Takeda et al. have shown that decreased activity of sarcolemmal Ca^2+^ pump occurs earlier compared to sarcoplasmic reticulum Ca^2+^ pump activity and myofibrillar Ca^2+^ stimulated ATPase activity [53]. The investigation by Golfman et al. supports this hypothesis as well [54]. In contrast, Sheikh et al. did not observe any significant changes in the plasma membrane Ca^2+^-ATPase activity during DCM, specifically in the cardiac endothelial cells [55]. It is possible that different cell types adapt differently during the DCM. These diverging results demonstrate the limiting ability of the cell to release Ca^2+^ through Ca^2+^ pump and Na^+^/Ca^2+^ exchanger in sarcolemma, therefore initiating the [Ca^2+^]_i_ overload contributing to detrimental cardiovascular outcomes.

### IId. Sarcolemmal membrane changes

As discussed previously, the EC coupling starts through LTCC of the sarcolemma following the entry of Ca^2+^. The sarcolemma can bind to the Ca^2+^ and thus regulate Ca^2+^ exchanges during EC coupling cycles. In the heart, Ca^2+^ binding pool of sarcolemma is linked with the residues of sialic acid [56]. The lack of sarcolemmal sialic acid content was shown to enhance Ca^2+^ exchange and may impair precise regulation of EC coupling cycles [57]. Thus, sarcolemmal Ca^2+^ binding ability might be critically important for the normal functioning of the heart. Accordingly, a pathophysiological modulation in the efficiency of this superficial Ca^2+^ pool could influence the mechanical performance of the heart. In diabetic cardiomyopathies, similar conclusion has also been reported by Pierce et al. [58, 59]. In hearts of diabetic rats, the level of sialic acid was significantly downregulated in myocardial sarcolemmal membrane, which was reversible by insulin therapy. Suppression in Ca^2+^ binding may be partly due to the lower content of neuraminidase-sensitive sialic acid residues, since neuraminidase treatment also failed to reduce the Ca^2+^ binding activity [58]. Therefore, sarcolemmal defect may contribute to the precise regulation of Ca^2+^ transits through the sarcolemma during diabetes and may lead to improper EC coupling.

### IIe. Maturation of cardiomyocytes

The normal functioning of the heart depends on a complex network of cells called cardiomyocytes, which exists in three-dimensional network of multiple cells and drive cardiac contractility. These cells are connected to the ECM produced by the supporting fibroblast cells, which transduces the force and coordinates with the contraction of the heart.

During maturation, the cardiomyocytes undergo several structural, metabolic, and physiological changes from conversion of fetal cardiomyocytes into the adult cardiomyocytes. Existing cardiomyocytes proliferate to regenerate the cardiomyocytes. Multiple factors are involved in progression of cardiomyocyte maturation [60, 61] but forced proliferation or maturation by inhibition/overexpression of cofactors, miRNA, molecules such as activated Yap (Yes-associated protein), cyclin B1-CDC2 complex, certain G1/S-phase molecules including CDK2, E2F1, cyclin D1 [62–64] may cause cardiac dysfunction. Therefore, it is very important to understand the tuning between proliferation and maturation to strategically design the parameters for enhancing cardiomyocyte regeneration and minimizing its side effects. The role of active cardiomyocytes is not well studied in the inflammatory responses underlying the DCM development and progression.

Diabetes-related inflammation induces mitochondrial dysfunction, impaired cardiomyocyte Ca^2+^ handling, oxidative stress, collagen-induced cardiomyocyte, and ECM stiffness. ECM accumulation at the cellular level in the heart leads to a cardiomyopathic phenotype resulting in heart failure with preserved ejection fraction (HFpEF) [65]. Moreover, homeostasis in myocardial tissue requires the balance between inflammatory damage and healing but diabetes mellitus promotes different inflammatory responses which further delays the healing process. Reduction in glycemic condition limits DCM and associated cardiac diseases as well. According to Tate et al., modification of normal glycemia with insulin reduced the collagen content, cardiomyocyte hypertrophy and controlled the progression of DCM in rats [66]. Additionally, abnormal expression of contractile and regulatory proteins contributes to impaired cardiac contraction. For example, phosphorylation of troponin is responsible for defective myocardial contractility since troponin and myosin both regulate the cardiomyocyte contraction [67].

Cardiomyocytes demand high energy due to its continuous contractions, which allows cells to utilize multiple substrates for energy production [68] in the heart. Previous reports suggests the reduction of glucose transporter (GLUT) 4 levels and depletion in glucose intake during hyperglycemia and insulin resistance [69]. The biopsies from the Type II diabetes patients also demonstrate significant reduction of GLUT4 and activation of PI3K/Akt signaling pathways at sarcolemma in diabetic mice and patients with non-insulin-dependent diabetes mellitus. Whereas, patients with left-ventricular dysfunction had limited activation of PI3K but not Akt and increased GLUT4 expression at sarcolemma [70].

Not only cardiomyocytes play a pivotal role in cardiac inflammation in DCM, cardiomyocytes exposed to excessive sugar/lipids generate a meta-inflammation like milieu as well [71]. The inflammasome activation is linked with the production of IL1β and IL18 which in turn induce the cardiomyocyte apoptosis [72].

### IIf. Transverse tubule (T-tubule)

T-tubules are highly branched invaginations of the sarcolemma in ventricular myocytes that are rich in ion channels and play critical role in EC coupling, signal transduction, initiation and regulation of action potential, and maintenance of resting membrane potential. T-tubules are critical for normal cardiac physiology and reported to be structurally and functionally compromised during disease. Disorganized/lost T-tubules has been shown in animal models of heart failure [73], this loss results in systolic and diastolic dysfunction, disrupted Ca^2+^ homeostasis leading to loss of contractility in failing myocardium [74, 75]. The subcellular mechanisms of dysfunction in DCM have been extensively investigated and numerous studies have linked alterations in T-tubule structure and function with cardiac disease etiopathologies [76]. Typically, the disease progression includes the reduced T-tubule density [77], T-tubule dilatation, loss of tubule opening at the cell surface, appearance of broad T-tubule sheets and changed orientation [78]. In a recent report it has been demonstrated that the density of T-tubules remain unchanged specifically in diabetic patients with HFpEF, while increased in non-diabetic controls with HFpEF, and decreased in heart failure with reduced ejection fraction (HFrEF). The T-tubules were found to be dilated in all the heart failure entities [79]. Moreover, the disruption in T-tubules promotes the asynchronous and slower Ca^2+^ absorption and release in rodents with HFrEF which resulted in diastolic dysfunction in HFpEF and HFrEF with diabetes.

Due to limited available data on T-tubule comparative studies in diabetic heart, a new confocal based laser scanning method has been used to examine the labelling of T-tubules, which showed a modest decrease in T-Power (also known as sarcomere power) in diabetic cardiomyocytes [80]. Another study by Setterberg et al. demonstrated the association of reduced T-tubule density with the asynchronous EC coupling in diabetic cardiomyocytes [81].

## III. Changes occurring in sarcoplasmic reticulum

The sarcoplasmic reticulum regulates [Ca^2+^]_i_ and cardiac contractility, as it participates in Ca^2+^ release, reuptake, and storage during the Ca^2+^ transits associated with the cardiac contraction-relaxation cycles. Each of these functions are achieved by three-special class of proteins: (1) cytosolic Ca^2+^ spikers/releasers: sarcoplasmic reticulum Ca^2+^ release channels (RyR2); (2) Ca^2+^storage by the luminal Ca^2+^-binding proteins (histidine-rich Ca^2+^-binding protein, calsequestrin (CSQ), sarcalumenin and junctate); and (3) Ca^2+^ uptakers: SERCA2a pumps for Ca^2+^ reuptake. As proper Ca^2+^ storage, release and reuptake are essential for normal cardiac function, any qualitative and quantitative changes in these regulators lead to impaired cardiac function. Several studies have shown that sarcoplasmic reticulum function is adversely affected during diabetes. For instance, sarcoplasmic reticulum-release and uptake activities play a crucial role in regulation of [Ca^2+^]_i_, which are decreased in the diabetic heart.

### IIIa. Sarcoplasmic reticulum Ca^2+^-ATPase (SERCA2a)

At the end of the EC coupling cycle, the [Ca^2+^]_i_ has to be reset to low levels to facilitate cardiac dilatation and ventricular filling. SERCA2a, the major isoform that is present in the cardiac tissue, plays a central role in pumping >70% of the sarcoplasmic Ca^2+^ back to the sarcoplasmic reticulum lumen. SERCA2a is negatively regulated by a peptide known as phospholamban (PLN). The PLN association with SERCA2a is determined by the phosphorylation status, which causes its dissociation from SERCA2a leading to higher transportation rate of Ca^2+^ through the pump. Classically, β-adrenergic stimulation is involved in enhancing the heart function through the PKA-mediated PLN phosphorylation [82]. Interestingly, SERCA2a also exists as a multimeric protein complex involving several regulators of SERCA2a function, which interacts either directly or indirectly with the SERCA2a [83].

Multiple defects in the SERCA2a expression and function were observed during diabetes. It has been demonstrated that reduced activity and expression of SERCA2a contributes to the reduced Ca^2+^ return into the sarcoplasmic reticulum, leading to early stage diastolic dysfunction during diabetes [84]. Such chronic reduction in Ca^2+^ returns lead to depletion of sarcoplasmic reticulum Ca^2+^ reserves resulting in systolic dysfunction and heart failure. It was further demonstrated that SERCA2a upregulation can ameliorate the diabetic cardiac dysfunction, which restores the Ca^2+^ transient to the normal levels [85]. Recent studies have provided several mechanistic insights regarding the diminished SERCA2a function during diabetes. Despite controversial reports on the SERCA2a levels and quantitative changes in the phosphorylation status of the PLN, it was consistently demonstrated that SERCA2a function was diminished in Type I and Type II diabetes mellitus [86]. There were some commonalities that exist in these two scenarios. For instance, AGE (Advanced glycation end products) and their receptor RAGE (Receptor for AGE) are highly upregulated in diabetic hearts and were found to modify SERCA2a [87]. Hence, it is most probable that AGE [88] can also alter SERCA2a interactions in such a way that SERCA2a function is compromised during diabetes. Alternatively, oxidative stress might downregulate SERCA2a expression through inactivation of its transcription factor, Sp1 [86]. In addition, high glucose levels might enhance O-GlcNAcylation of Sp1 transcription factor, which is the crucial regulator of SERCA2a gene, ATP2A2, in the heart. Such alteration in Sp1 was shown to reduce its transcriptional activity. Furthermore, enhanced O-GlcNAcylation of PLN can also enhance its association with SERCA2a leading to inhibition [86]. Recently, a role for histone acetylation was noted in regulation of SERCA2a expression [89]. Given that HDACs contribute to adverse diabetic cardiac remodeling [90], it is plausible that there might be involvement of epigenetic regulation in SERCA2a expression. Additionally, a recent report has shown that cardiac-specific deletion in PKBα/β inhibits the insulin-dependent phosphorylation of striated muscle preferentially expressed protein kinase (SPEG) and SERCA2a inducing Ca^2+^ re-uptake by sarcoplasmic reticulum leading to cardiac dysfunction [91]. In summary, based on various assessments and studies, diabetes reduces SERCA2a function and activity at both cellular and protein levels and alters its interactions with its regulators leading to DCM.

### IIIb. Ryanodine receptor 2 (RYR2)

RYR2 is a Ca^2+^ releasing protein present in the sarcoplasmic reticulum of the cardiomyocytes and is responsible for the Ca^2+^ sparks following the Ca^2+^ entry into the cytosol following membrane depolarization. Upon binding with the Ca^2+^ and entering the cytosol as part of Ca^2+^ currents, RYR2 is responsible for the Ca^2+^ sparks, known as CICR, which determines the extent of cardiac contraction. Thus, the extent of Ca^2+^ spike is dependent on the RYR2 function as well as the Ca^2+^ stores in the endoplasmic reticulum. As RYR2 function is critical in determining the free Ca^2+^ spikes in the cytosol, any disruptions in the RYR2 levels and/or function are associated with the diminished cardiac contractile response. In fact, diabetes has been reported to cause changes in RYR2 function leading to contractile dysfunction. During the early stages of dysfunction, there were arrhythmias owing to the Ca^2+^ leak from the sarcoplasmic reticulum and during the later stages, contractile dysfunction owing to the depleted RYR2 levels. Studies in the mouse models simulating cardiac lipid overload (a frequently noted abnormal shift to enhanced fatty acid oxidation, observed in the Type II diabetes mellitus patient’s heart) have noted that enhanced mitochondrial oxidative stress and lipid overload leads to RYR2 oxidation leading to Ca^2+^ leak and arrhythmias [92]. Interestingly, other studies also emphasized the significance of oxidative stress in cardiac arrhythmias and demonstrated that glucose intolerance leads to inappropriately enhanced Ca^2+^/calmodulin-dependent protein kinase II (CaMKII) mediated phosphorylation-dependent activation of RYR2, which is also dependent on the oxidative stress leading to Ca^2+^ leakage [93, 94]. During the later stages, depletion of the RYR2 levels and its stabilizer FKBP12.6 were noted, which potentially could lead to Ca^2+^ release during diastole, leading to abnormal contractility and Ca^2+^ loss [95–97]. Although much is known about the RYR2 interactors, less is known about the mechanisms of its downregulation and altered interactions during diabetes [98].

###  IIIc. Ca^2+^ binding proteins

The main Ca^2+^ binding/storage protein in the cardiac sarcoplasmic reticulum is the CSQ [99]. Interestingly, it was noted that diabetes-induced cardiac dysfunction also involves reduced levels of CSQ, and rescue of the CSQ levels were associated with the amelioration of the dysfunction [100]. However, others have not observed such changes in the cardiac tissue [101]. Nonetheless, CSQ polymorphisms were suggested to influence the risk of Type II diabetes mellitus in certain human populations [102]. Further studies are needed to demonstrate the relationship between CSQ and its role in DCM.

## IV. Epicardial adipose tissue (EAT)

Epicardial adipose tissue (EAT) is a multifaceted fat depot that confers the mechanical protection to the coronary arteries from distortion and compression during the excitation and contraction of the myocardium [103–105]. EAT displays a higher rate of lipogenesis and fatty acid metabolism which are critical for the proper functioning of heart. EAT related functional and morphological changes are age and disease specific [106]. Recent evidence has shown that increased levels of EAT can induce various pathologies and can alter the Ca^2+^ handling that eventually lead to contractile dysfunction of cardiomyocytes [107]. Greulich et al. have reported the reduced contractility and Ca^2+^ activity in the cardiomyocytes isolated from the animals eating a high fat diet, as compared to the animals on normal diet [108]. Although the metabolic crosstalk between EAT and the Ca^2+^signaling in context of DCM is poorly understood, the existence of compelling evidence suggests the importance of correct functioning of EAT is required for proper Ca^2+^ signaling and cardiac activity. Thus, these interactions between EAT and Ca^2+^ signaling should be investigated on a larger scale to identify clinically relevant molecules that might uncover the novel pharmacologic interventions for the treatment of DCM.

## V. Changes occurring in mitochondria

Although mitochondrial dysfunction has been implicated in the DCM nearly three decades ago, underlying mechanisms of functional and structural changes associated with DCM are not fully understood [109, 110]. The main features of such dysfunction include reduced energy production and excessive generation of reactive oxygen species (ROS). Interestingly, mitochondrial Ca^2+^ load influences these two interlinked processes to a certain extent. Optimal levels of mitochondrial Ca^2+^ leads to enhanced metabolism and ATP production leading to reduced ROS production [111]. However, Ca^2+^ overload leads to abnormal mitochondrial permeability transition pore (MPTP) opening, thereby reducing ATP production culminating in enhanced ROS production. Interestingly, excessive ROS production also leads to enhanced mitochondrial permeability leading to Ca^2+^ overload [112]. Excessive ROS also leads to [Ca^2+^]_i_ overload through Ca^2+^ leak from RYR2 and reduced cellular efflux [113]. As ROS and Ca^2+^ overload can regulate each other in a positive reciprocal fashion and both can adversely open MPTP independently, it has been postulated that during diabetes and ischemic conditions, excessive ROS and mitochondrial Ca^2+^ overload jointly trigger mitochondrial death [111]. Consistent with this hypothesis, reduced ATP production in diabetic hearts was observed, presumably due to dysfunctional mitochondria. These findings suggest that mitochondrial Ca^2+^ load acts as a sensor of Ca^2+^ homeostasis in the cell, thereby triggering the pathological outcomes associated with diabetes. Diabetic cardiac mitochondrial dysfunction and reduced productivity also involves extensive remodeling of mitochondrial structure, lipid and protein composition. This has been evident in two different animal models of diabetes [114–116]. Although the abnormal remodeling in the mitochondria is well appreciated, the underlying cause for such changes and the possibility of reversal of these changes need to be established. Nonetheless, most of these changes might have occurred due to the sustained damage inflicted by the enhanced Ca^2+^ overload and ROS. It is interesting to note that certain degree of uncoupling of mitochondrial oxidative phosphorylation exists in different types of diabetes and ROS has been implicated in such uncoupling adaptations [117, 118]. Furthermore, in the animal models of diabetes, inducing exercise was shown to reverse cardiac mitochondrial dynamics to certain extent [119]. Also, others have reported that exercise enhances antioxidant capacity and potentially prevent mitochondrial Ca^2+^ overload, enhanced endoplasmic reticulum Ca^2+^ uptake and reduced endoplasmic reticulum Ca^2+^ leak [120]. In this context, it is of great significance to develop exercise mimetics to better translate exercise-induced diabetic cardiac benefits, especially for advanced DCM patients.

In addition to the above findings, the mitochondrial Ca^2+^depletion was also proposed to cause mitochondrial defects leading to DCM. The mitochondrial Ca^2+^ uptake is mainly regulated by the MCU. The MCU activity is highly tissue-specific, which is tightly controlled by the active cells with intensive cytosolic Ca^2+^ signaling required for the integrity of the mitochondria [121]. Among others, one of the physiological roles of MCU complex is controlling the ATP production through activation of Ca^2+^-dependent dehydrogenase in the mitochondrial matrix, the manipulation in any of the MCU components could alter the pyruvate dehydrogenase activity and intracellular ATP levels in various human cells such as HeLa cells [122], and pancreatic β cells [123]. Alteration of MCU complex could also modulate the cellular metabolism [124], its presence and/or absence also controls the cell death [125, 126]. The inhibition and/or overexpression of MCU leads to abnormal pathophysiological disease states such as [127] reduced cardiac performance and enhanced energy demand [128, 129], alter the beat-to-beat amplitude of cytoplasmic Ca^2+^ oscillations [130]. It has been noted that higher glucose levels was associated with reduced MCU levels, which might be the reason behind the reduced mitochondrial Ca^2+^ levels [131]. Furthermore, it was shown that restoration of the MCU levels in the cardiomyocytes resulted in amelioration of mitochondrial metabolic deficiencies and heightened oxidative stress induced by high glucose levels [131]. Alternatively, enhanced mitochondrial Ca^2+^efflux might also lead to mitochondrial Ca^2+^depletion most likely due to increased Na^+^ levels (as discussed below). Although further understanding is necessary, it is possible that both mitochondrial Ca^2+^ depletion and overload might be occurring at different stages of diabetes, the former in the early stages and the later in the late stages of diabetes.

## VI. Changes occurring in the myofibrils

Myofibrils are the structural units responsible for contraction and relaxation cycles and occupy more than half of the total volume of myocardial cells. The myofibrils participate at the end stage of the EC coupling process and are made of thin (actin) and thick (myosin) filaments apart from other regulatory and supportive proteins such as tropomyosin (TM), troponins, and titin. The major function of cardiomyocyte involves cyclic contraction and relaxation that is tightly regulated by complex interaction of contractile proteins and different membrane proteins in the heart, a process known as EC coupling [132]. During EC coupling following stimulation, the release of Ca^2+^ from sarcoplasmic reticulum to the myofilament and binds to troponin C, for a conformational change in the location of TM on actin, thereby exposing the myosin-binding site. During muscle relaxation, TM blocks the myosin-binding site on actin when the cytoplasmic levels of Ca^2+^ is low. It has been demonstrated that the expression of myofibrillar proteins and their isoforms is highly regulated and dynamically changed depending on the age, species, physiological and pathological conditions, including diabetes mellitus [133–135]. Previous reports confirm the relationship between myosin ATPase activity, the maximum velocity of shortening, myosin isoenzyme composition and speed of cardiac muscle shortening in rat hearts [136, 137].

Two main features of diabetic hearts with regard to the functional decline include- reduced Ca^2+^ sensing ability of the regulatory proteins of the actomyosin system and myosin isoform shift [67]. The decline or lack of MHC was associated with decline in functional efficiency [67, 138]. Some of these changes (enhanced isoform switching from the normally expressed α-MHC to the fetal isoform β-MHC) can be noticed even before the overt diabetic cardiac phenotype [139]. Perhaps, in rodents, this isotype switching could be an adaptive mechanism in response to the mitochondrial dysfunction and lower ATP production, as these isoforms, α-MHC and β-MHC generate same force, but the former consumes more ATP [133, 140]. The regulatory mechanism of MHC isoform switch in diabetes might be similar to that of thyroid dysfunction [141]; in diabetes and hypothyroid state, the antisense RNA and α-MHC transcription are turned-off. Insulin treatment in different animal models was shown to reverse the decreased myofibrillar ATPase activity and MHC isoform changes [142].

Apart from changes in the isotype switching, alterations in posttranslational modifications of the regulatory proteins of actomyosin formation have been involved in cardiac dysfunction during diabetes. The activities of depressed myofibrillar ATPase may be related to changes in the cardiac troponin I subunits of diabetic hearts, as cardiac troponin I  phosphorylation is reported to modify the ATPase activity. Previous investigations suggests the increase (by 40%) in cardiac troponin I phosphorylation in the diabetic hearts, which can be reversed by administration of insulin [143]. Furthermore, PKC mediated cardiac troponin I phosphorylation was shown to reduce Ca^2+^ sensitivity and force generation, which is higher during diabetes due to [Ca^2+^]_i_ depletion and enhanced PKC phosphorylation [144–146]. Like cardiac troponin I, phosphorylation changes in myosin light chain (MLC) protein may also have a modulatory role in altering the myofibrillar ATPase activity [147]. In this regard, Liu et al. [147] reported that in the diabetic rat heart, the protein contents of MLC, MLC-kinase and MLC phosphorylation were significantly decreased (40% to 45% and 30% to 45%, respectively), and insulin administration can reverse these changes. Phosphorylation of MLC at a regulatory site near to the binding domain of calmodulin increased the concentration of Ca^2+^/calmodulin required for MLC kinase activity, whereas non-phosphorylated MLC have opposite effect in diabetic heart, which may partially explain the reason of decreased myofibrillar ATPase activity and impaired contractile function [148]. All these observations highlight the central role of myofibrillar protein changes and their post-translational modifications in diabetes and thus revealing their contribution in decreased cardiac contractility.

## VII. Role of  Na^+^ in Ca^2+^ signaling

Intracellular Na^+^ levels not only regulate osmotic strength, but also contribute to the net positivity of the cell. Because of these reasons, cells use Na^+^ flux to regulate Ca^2+^ transits, often in an opposite direction. Recent report suggests that increased dependency of the diabetic hearts on the Na^+^-glucose cotransporter (SGLT) for glucose uptake resulted in Na^+^ overload in the hearts, which has been postulated to contribute to the arrhythmia and enhanced oxidative stress [149]. In another study, it was found that Na^+^/H^+^ exchanger could potentially contribute to the Na^+^ overload in the presence of high glucose levels [150]. While elevated levels/function of the SGLT and Na^+^/H^+^ exchanger are the main cause for Na^+^ overload during Type II diabetes, the decreased activity of Na^+^/K^+^ pump and Na^+^/Ca^2+^ exchanger contributes to the Na^+^ overload during Type I diabetes [150, 151]. Nonetheless, the downstream adverse effects of Na^+^ overload seem to be common in these models of diabetes. 

The enhanced intracellular Na^+^ levels/Na^+^ overload might cause efflux of Ca^2+^ from the mitochondria through the mitochondrial Na^+^/Ca^2+^ exchanger and thus may deprive the Ca^2+^ mediated enhancement in oxidative phosphorylation. It has been known that moderate increments in the mitochondrial Ca^2+^ can enhance activities of dehydrogenases and ATP synthase culminating in enhanced ATP production [152]. Hence, Na^+^ overload might cause cardiac contractile dysfunction through reduced mitochondrial Ca^2+^ levels and ATP production.

## VIII. The effect of inflammation on Ca^2+^ signaling

The landmark studies correlating inflammation with diabetes were conducted in early 90’s by Hotamisligil group, who reported the critical role of TNF-α in obesity and Type II diabetes [153]. Following this, several investigators have studied inflammation in relation to Type II diabetes by measuring the circulating concentrations of inflammatory markers/mediators [154]. Studies conducted in human and animal models in the past decade have supported this correlation by providing further evidences for the role of inflammation in initiation, development and progression of diabetes [155]. A recent study has shown association of activated pro-inflammatory pathways in response to insulin action with obesity and other metabolic disorders including Type II diabetes [156]. Inflamed β cell pancreatic islets also known as insulitis is a characteristic feature of Type I diabetes [157]. A study by Anderson et al., suggests the failure of central and peripheral immune tolerance results in the activation of autoreactive T cells in diabetic mice [157]. Involvement of potential inflammatory pathways in pathophysiology of diabetes has advanced the interest of targeting inflammation/inflammatory biomarkers for the prevention and control of diabetes .

Cardiac inflammation contributes to cardiomyocyte loss, fibrosis, and dysfunction leading to DCM [24, 158]. Various molecular mechanisms associated with diabetic myocardial inflammation is related to activation of NF-κB signaling pathway and the renin–angiotensin–aldosterone system [159]. Several neurohormones and pro-inflammatory molecules, such as IL6 and IL8, TNF-α, monocyte chemotactic protein 1 (MCP1), adhesion molecule intercellular adhesion molecule 1 (ICAM1), and vascular cell adhesion molecule 1 (VCAM1), actively contribute to the myocardial oxidative stress, fibrosis, and cardiac dysfunction [11, 160, 161]. The increased levels of these inflammatory responses in the heart have been shown to directly influence cardiac function through multiple mechanisms. The resulting inflammatory mediators alter intracellular signaling mechanisms in cardiomyocytes for the development of DCM. Myocardial overexpression of TNF-α transgenic mice resulted in heart failure due to Ca^2+^ handling defects and cardiac dilation [160].

Inflammatory cytokines such as TNF-α and IL1β has shown to decrease the expression of Ca^2+^-regulating genes (SERCA2a and Ca^2+^ release channel) thus leading to a negative ionotropic effect due to modification in [Ca^2+^]_i_ homeostasis in adult cardiomyocytes [162]. Abnormalities in sarcoplasmic reticulum Ca^2+^ release promote myocardial remodeling (hypertrophy, substantial fibrosis, ventricular dilation, pump failure) resulting in heart failure due to pressure overload. These findings demonstrate that inflammation-triggered Ca^2+^ imbalance can contribute to cardiac remodeling [163]. Acute exposure to IL1β caused NLRP3-signaling activation and CaMKII-dependent RyR2/PLN hyperphosphorylation and enhanced spontaneous sarcoplasmic reticulum Ca^2+^-release events in both postoperative atrial fibrillation cardiomyocytes and HL-1-cardiomyocytes [164]. Previous studies have also shown that murine Ca^2+^-sensing receptor (CASR) activates the NLRP3 inflammasome which is mediated by the increase in [Ca^2+^]_i_ and decrease in cellular cyclic AMP [165]. The pressure overload activates the CaMKIIδ which in turn triggers the inflammatory gene response and activates the NLRP3 inflammasome in cardiomyocytes [166]. Recently, TNF-α signaling pathway in cardiomyocytes were associated with Ca^2+^ signaling in Type II diabetes obese (db/db) mice. TNF-α upregulated transient [Ca^2+^]_i_ amplitude and expedited its decay without changing the sarcoplasmic reticulum Ca^2+^ load or its spark frequency in mice [167]. Also, alteration of Ca^2+^ signaling and TNF-α, is gender specific, displaying the increase in TNF-α cardio-protective effect in male mice.

On the other hand, it is not clear whether Ca^2+^ signaling affects inflammatory response. Previous report has shown that the regulation of cell proliferation, energy, cell death of T-cells [168] and different steps of the inflammatory responses are associated with active Ca^2+^ [169–172]. CAMKII is known as the key regulator of the generation of inflammatory response, as it can act as a [Ca^2+^]_i_ sensor [173–175]. Cardiac stress activates the NF-κB-dependent inflammatory transcription pathway and oxidative injury, whereas the CaMKII is triggered by oxidation [176]. Singh et al. has also shown the association of increased CAMKII activity and inflammation in heart failure [177]. The increased levels of TNF-α during ischemia/reperfusion injury is related to Ca^2+^ overload resulting in cardiac dysfunction [178, 179]. The different forms of CAMKII have been shown to display distinct functions such as Ca^2+^-independent form can enhance the formation of T-cells and help in modulation of cell death [180]. CaMKII regulates the production of IL2, IL4, and IL10 by T-lymphocytes, which is also involved in the Ca^2+^-dependent IL2 transcriptional arrest [174, 181]. Studies on macrophages suggested role of CAMKII as a booster of pro-inflammatory cytokines and production of interferons on stimulation with toll-like receptors (TLRs)  [182]. Moreover, the previous studies suggests that G protein-coupled CaSR-dependent inflammation activates the NLRP3 inflammasome that induces the maturation and secretion of IL1β but the inhibition of ERK pathway reduced the activation of CaSR-dependent NLRP3 inflammasome [183]. Increased levels of Ca^2+^ through CaSR can stimulate multiprotein inflammasomes which helps in the maturation of proinflammatory cytokine IL1β through Caspase-1, making CaSR a promoter and responder of inflammation [184].

In vascular smooth muscle cells, angiotensin II induces the activation of NLRP3 inflammasome associated with CaSR and collagen synthesis, the inhibition of both CaSR and NLRP3 inflammasome promotes the secretion of proinflammatory cytokines [185]. Additionally, in monocytes and macrophages, CaSR can activate the NLRP3 inflammasome mediated by the increase in [Ca^2+^]_i_ [186].

## IX. Epigenetic regulation of Ca^2+^ mediated processes

Epigenetic mechanisms such as histone modification and DNA methylation regulate gene expression and play an important role in different cellular processes.

Histone modification has a major impact on chromatin structure and gene expression. Of the several types, histone acetylation is the most widely studied and robustly associated modification. It is regulated by histone acetyl transferases (HATs) and HDACs. HDACs are known as the key modulators that controls the proteostasis by changing the acetylation status; altered proteostasis has been studied in cardiovascular diseases including hypertrophy, heart failure in the past [187, 188]. According to a study by Chen et al. in diabetic mice, reduction in HDACs attenuated the cardiac hypertrophy and fibrosis in diabetic heart disease and inhibited the apoptosis by increasing the GLUT1 acetylation and decreased Caspase-3 activity [189]. Whereas in another study, increased HDAC levels led to myocardial ischemia and reduced mitochondrial dysfunction in diabetic heart [190]. Recent study showed the role of HDAC in regulation of Na^+^/Ca^2+^ exchanger, which is responsible for Ca^2+^ flux and efflux in cardiomyocytes [191, 192]. In porcine model of heart failure, the downregulation of HDAC affected the potassium channels [193].

DNA methylation regulates gene expression by altering the DNA stability, chromosomal structure, and DNA conformation. It works in proximity of histone modifications and miRNA to regulate the transcription. DNA methylation is catalyzed by a set of DNA methyltransferases (DNMTs) and previous studies suggest an important role of DNMTs in maintaining the homeostasis of cardiomyocytes in normal and stressed conditions [194]. In a study by Kumar et al., decreased Sirtuin 1 (SIRT1) and DNMT3b activity could increase the levels of histone H3 acetylation and CpG demethylation in diabetes-induced oxidative stress [195] and ROS-mediated stress, which ultimately lead to myocardial inflammation [196]. Reduced global methylation and increased hypomethylation have been previously associated with the development of atherosclerosis and other cardiac complications [197–199].

Growing evidence supports the hypothesis that during diabetes, the heart goes through epigenetic reprogramming. Although much has not been studied about these processes in pathogenesis of DCM, emerging evidence suggests that they might play a crucial role. Once identified, these epigenetic regulatory mechanisms could act as a potential target for drug discovery but detailed elucidation of these mechanisms in pathogenesis/manifestation of DCM needs further elucidation.

## X. Exosome and micro-RNA regulation of Ca^2+^ dynamics

Exosomes are nanosized extracellular vesicles (40–140 nm in diameter) that originate from multivesicular bodies, secreted by different cells, and found in body fluids such as plasma, saliva, urine, and serum. Exosomes play crucial role in intercellular communication by promoting the transport of macromolecules such as miRNA, noncoding RNA, DNA, lipids and proteins between the cells [200, 201].

Tissue microenvironment, including diabetes-induced effects modulate exosome cargo and has been shown to regulate communication between various cardiac cells (cardiomyocytes, fibroblasts, and endothelial cells) and among the heart and peripheral tissues/organs such as bone marrow, lungs, vasculature, kidney, and immune cells [202]. Our recent report demonstrates that under diabetic conditions, macrophage-secreted exosomes are enriched in HuR (mRNA-stabilizing protein) which activates profibrogenetic response in the heart [203]. miRNA has emerged as a key regulator of gene expression at the post-transcriptional level and has been shown to regulate several cardiac pathologic changes [204–206]. Different reports suggest the role of miRNAs in controlling the gene expression of certain inflammatory cytokines, Ca^2+^ handling and signaling proteins [46, 207]. Yildirim et al. found the upregulated expression of muscle-specific miRNA-1 in diabetic heart due to its binding to [208] molecular target Junctin (a key component of RyR2 Ca^2+^ release channel complex). Previous studies have already shown the role of miRNA-1 in impairing the cardiac relaxation and induction of cardiac hypertrophy and arrhythmia [209, 210]. Wahlquist et al. has reported that use of miRNA-25 impairs the Ca^2+^ uptake and aggravates cardiac dysfunction by interacting with SERCA2a [211]. The overexpression and inhibition of miRNA-1 influences Ca^2+^ flux in cardiomyocytes [212]. The increase in [Ca^2+^]_i_ concentration directly upregulates the expression of certain apoptotic genes. miRNA-145 could inhibit the Ca^2+^ overload and the overexpression of miRNA-145 has a protective effect against the ROS-induced cardiomyocyte apoptosis [213, 214]. miRNA-25 [38], miRNA-1, miRNA-138, miRNA-133a and miRNA-214 have been shown to influence mitochondrial Ca^2+^ homeostasis [213]. Macrophage-derived miRNA-155 promotes cardiac inflammation by inducing the secretion of inflammatory cytokines such as IL1β, IL6 and TNF-α [215]. It is plausible that exosomes carrying this miRNA might alter Ca^2+^ signaling in target cells like cardiomyocytes in DCM. However, very little is known about such a possibility. Mayourian et al. [216], reported that MSC-derived exosomal miRNA-21-5p could improve the contractile force of ischemic cardiomyocytes by regulating the Ca^2+^ homeostasis [216]. Also, it is not known so far if Ca^2+^ signaling related proteins or transcripts are enriched in exosomes under pathologic conditions. Interestingly, Ca^2+^ regulates multivesicular body formation and exosome release. For example, increased Munc13-4 (Ca^2+^-dependent Rab binding protein) is associated with increased Ca^2+^ uptake, multivesicular body formation and exosome release [217]. Whether DCM-induced alterations in Ca^2+^ signaling could impact exosome biogenesis, release and cellular uptake needs further investigation.

## XI. Therapeutic agents targeting Ca^2 +^ dynamics in DCM

As this review clearly focusses on the importance of Ca^2+^ handling proteins in the development of hypertrophy and heart failure in DCM, we summarize here some of the novel therapeutic approaches targeting Ca^2+^ handling proteins in modulating this aberrant expression and activity to improve symptoms in patients with DCM.

By modulating expression of Ca^2+^ handling proteins, gene therapy targeting RyR2, SERCA2a and PLN is gaining fresh impetus recently, as one of the modalities to treat contractile dysfunction in DCM. S100A1 is a Ca^2+^ binding protein, which modulates RyR2 and SERCA2a activity [218]. In animal models of acute and chronic ischemic heart failure, adenoviral-based delivery of S100A1 has shown to improve cardiac function by restoring Ca^2+^ homeostasis [219–223]. Like this approach, several studies have explored the use of adenoviral delivery of AC6 in the setting of congestive heart failure as well. AC6 has shown to downregulate the expression of PLN [224]. In a pig model of heart failure, adenoviral delivery of AC6 mitigated adverse ventricular remodeling and improved cardiac function [225]. Following these promising results, Hammond et al. and Penny et al. conducted a randomized controlled clinical trial in 56 HFrEF patients and demonstrated improvement in ejection fraction 4 weeks after the delivery of AC6 adenoviral vector [ClinicalTrials.gov identifier NCT00787059] [226, 227]. Although both S100A1 and AC6 delivered viral vectors have shown beneficial effects in animal models and early clinical trials, gene therapy using these vectors requires further elucidation in large multi-center clinical trials. Additionally, direct gene transfer using adenoviral-associated SERCA2a has also shown to improve Ca^2+^ transients and cardiac function in animal models of heart failure [228–231]. Given that SERCA2a gene transfer improved outcomes in animal models of heart failure, AAV1.SERCA2a was administered in Phase I and Phase II clinical trials [ClincalTrials.gov identifiers NCT00534703 and NCT00454818] [232–234]. The results of the initial Phase I and IIa trials suggested that AAV1.SERCA2a treatment reduced the number of cardiac events in heart failure patients [232, 235]. With these initial promising results, clinical trials are using AAV1.SERCA2a vector, istaroxime, a dual functional lusoinotropic agent which stimulates SERCA2a and inhibits Na^+^/K^+^ ATPase [236]. Also, by inhibiting PLN-mediated SERCA2a activity through cAMP/PKA-dependent mechanism, istaroxime has shown to increase Ca^2+^ uptake into the SR [237]. Animal studies in heart failure have shown improvement in cardiac function using I.V. administration of istaroxime [238, 239]. Additionally, early clinical studies and HORIZON-HF concurred beneficial effects on cardiac contractility and showed reduction in diastolic stiffness in heart failure patients using istaroxime [240, 241]. Large scale clinical trials for istaroxime are currently on-going [ClincalTrials.gov identifiers NCT02617446 and NCT02477449].

Another interesting approach is by modifying SERCA2a and PLN using nitroxyl (HNO/NO−). BMS-986231 has undergone extensive clinical testing using HNO derivative. Early clinical trials have demonstrated BMS-986231 to be safe and efficacious in HFrEF patients [242] [ClincalTrials.gov identifier NCT02157506]. Based on these promising results, currently, 3 clinical trials are on-going to address the efficacy and safety of BMS-986231 in patients with various forms of heart failure [ClincalTrials.gov identifiers NCT03016325, NCT03357731, and NCT03730961] [243].

## Conclusions

**Fig. 2:**
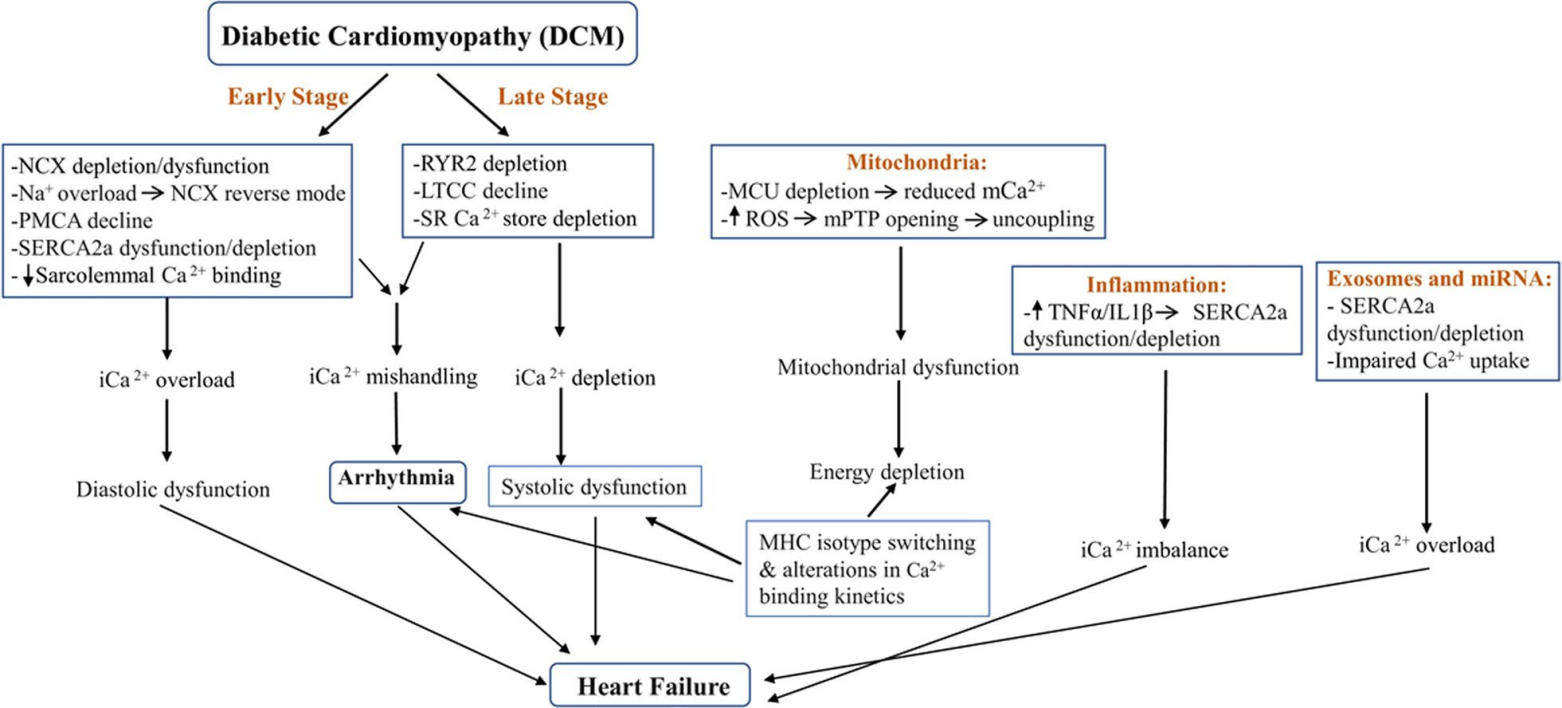
Salient molecular changes in the regulators of the Ca^2+^ transits during excitation-contraction (EC) coupling associated with DCM. Arbitrary separations of the early and late-stage changes are also indicated. These changes eventually lead to the diabetic cardiomyopathic phenotypes, mitochondrial changes, inflammation, and exosome mediated effects on Ca^2+^ transits leading to heart failure. *NCX* N^+^/Ca^2+^ exchanger; *PMCA* Sarcolemmal/plasma membrane Ca2^+^-ATPase; *SERCA2a* Sarco(endo)plasmic reticulum Ca^2+^-ATPase; *RYR2* Ryanodine receptor 2; *LTCC* L-type calcium channels; *SR* Sarcoplasmic reticulum; *MCU* Mitochondrial Ca^2+^uniporter; *mCa*^*2+*^ mitochondrial Ca^2+^; *ROS* Reactive oxygen species; *mPTP* Mitochondrial permeability transition pore; *iCa*^*2+*^ Intracellular Ca^2+^; *MHC* Myosin heavy chain

DCM involves failure of Ca^2+^ handling at multiple transports and ion channels located at sarcolemma, sarcoplasmic reticulum, and mitochondria (Fig. 2). Though there are discrepancies regarding the level of involvement in each of the above discussed Ca^2+^ transit regulators, it is evident that Ca^2+^ mishandling remains the major cause in the development of DCM. Inflammatory mediators and miRNAs are shown to interact with regulators of Ca^2+^ signaling as well. Some of these miRNAs and inflammatory mediators are shown to be enriched in exosomes, however, it’s not clear if transcripts/proteins directly related to Ca^2+^ signaling are modulated in exosomes during DCM pathogenesis. Reversal of certain changes (for example SERCA2a over-expression) has been proven to be beneficial in ameliorating DCM. However, to find wide applicability, it is necessary to develop highly selective modulators to correct aberrant cardiomyocyte Ca^2+^ transits and Ca^2+^ handling proteins.

## Data Availability

Not applicable.

## Abbreviations

Ca^2+^: Calcium
[Ca^2+^]i: Intracellular calcium concentrations
CICR: Calcium-induced calcium release
MCU: Mitochondrial Ca^2+^ uniporter
DCM: Diabetic cardiomyopathy
EC coupling: Excitation–contraction coupling
ECM: Extracellular matrix
RyR2: Ryanodine receptor 2
SERCA2a: Sarco(endo)plasmic reticulum Ca^2+^-ATPase
SPEG: Striated muscle preferentially expressed protein kinase
EAT: Epicardial adipose tissue
PLN: Phospholamban
TM: Tropomyosin
HATs: Histone acetyl transferases
HDACs: Histone deacetylases
DNMTs: DNA methyltransferases
MHC: Myosin heavy chain
Na^+^: Sodium
SGLT: Na^+^-glucose cotransporter
POAF: Postoperative atrial fibrillation
CASR: Ca^2+^-sensing receptor
CaMK: Ca^2+^/calmodulin-dependent protein kinase
VSMC: Vascular smooth muscle cell
Ang II: Angiotensin II
HFpEF: Heart failure with preserved ejection fraction
HFrEF: Heart failure with reduced ejection fraction
Yap: Yes-associated protein
CSQ: Calsequestrin
MPTP: Mitochondrial permeability transition pore
Cav3: Caveolin-3
LTCC: L-type calcium channels
CSQ: Calsequestrin
TNF-α: Tumor necrosis factor-α
MCP1: Monocyte chemotactic protein-1
ICAM1: Adhesion molecule intercellular adhesion molecule-1
VCAM1: Vascular cell adhesion molecule-1
CASR: Ca^2+^-sensing receptor
miRNA: microRNA
AGE: Advanced glycation end products
RAGE: Receptor for advanced glycation end products
Hsp20: Heat shock protein 20
GLUT: Glucose transporter
SIRT1: Sirtuin 1
ROS: Reactive oxygen species
MLC: Myosin light chain
TLR: Toll-like receptor
TSG101: Tumor Susceptibility 101
